# Trastuzumab-associated prurigo nodularis successfully treated with nemolizumab: A case report

**DOI:** 10.1016/j.jdcr.2025.10.046

**Published:** 2025-11-06

**Authors:** Ryan Chen, Michelle S. Lee, Ashleigh Eberly Puleo, Arshna Qureshi, Alvaro C. Laga, Vinod E. Nambudiri

**Affiliations:** a Department of Dermatology, Brigham and Women’s Hospital, Boston, Massachusetts; b University of Massachusetts Chan Medical School, Worcester, Massachusetts; c Harvard Medical School, Boston, Massachusetts; d Dana Farber Cancer Institute, Boston, Massachusetts; e Department of Pathology, Brigham and Women’s Hospital, Boston, Massachusetts

**Keywords:** chronic pruritus, drug-induced skin eruption, nemolizumab, prurigo nodularis, trastuzumab

## Background

Prurigo nodularis (PN) is a chronic inflammatory dermatosis marked by intensely pruritic, hyperkeratotic, or excoriated papules and nodules.^1^ Recent advances in its pathogenesis highlight the role of neuroimmune pathways, especially interleukin-31 (IL-31) signaling, in mediating pruritus.^2^ In addition to cytokine dysregulation, PN is associated with increased dermal nerve fiber density and elevated neuropeptides such as substance P and calcitonin gene-related peptide, which may amplify itch through neurogenic inflammation.^1^ Nemolizumab is a first-in-class monoclonal antibody targeting the IL-31 receptor A that was FDA-approved for treating PN after demonstrating efficacy in phase 3 clinical trials.^3^

Trastuzumab, a humanized monoclonal antibody targeting HER2, is a cornerstone in the management of HER2-positive breast cancer.^4^ Pruritus is a recognized adverse event associated with HER2 blockade that can persist even after discontinuing the inciting agent.^5^^,^^6^ While trastuzumab has been associated with generalized pruritus in clinical trials, with reported rates ranging from 11% to 17.6%, the development of PN secondary to trastuzumab has not been described.^6^ We report a novel case of trastuzumab-induced pruritus and PN refractory to conventional therapies and dupilumab, which ultimately responded to targeted treatment with nemolizumab.

## Case report

**Fig 1:**
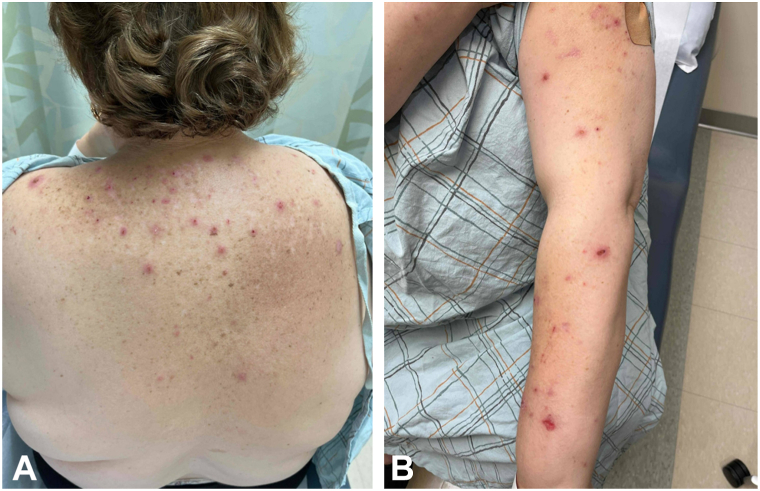
On initial presentation, the patient had **(A)** scattered erythematous excoriated papules and nodules on the back **(B)** and erythematous excoriated to eroded papules and plaques on the left upper extremity.

A 60-year-old diabetic female with HER2-positive invasive ductal carcinoma of the right breast (pT1cN1) presented with severe, generalized pruritus shortly after initiation of adjuvant paclitaxel, trastuzumab, and pertuzumab. She developed intermittent but intense pruritus predominantly affecting her upper back, shoulders, arms, and legs with formation of associated excoriations and papulonodules (Fig 1). She also developed facial pruritus, primarily on the left side, which she partially attributed to anxiety. Her pruritus became severe enough to disrupt her sleep and significantly affected her quality of life. She presented to the emergency room and was prescribed hydroxyzine, which paradoxically worsened her symptoms. She denied prior dermatologic conditions or known triggers.

**Fig 2:**
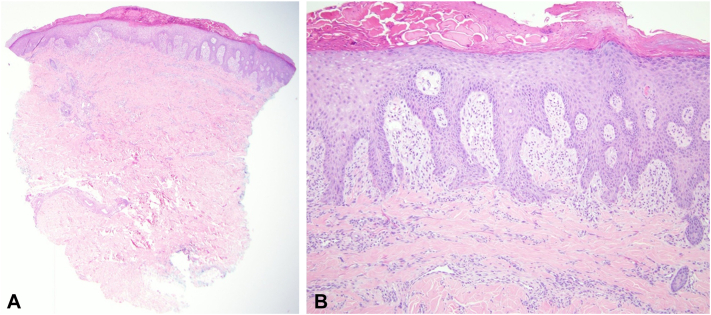
Histologic features of the left upper arm punch biopsy. **A,** Epidermal hyperplasia with thick scale. **B,** Irregular elongation of the rete ridges with overlying scale crust, adjacent compact hyperkeratosis, hypergranulosis, and associated papillary dermal fibrosis.

Evaluation revealed no evidence of anemia, thyroid dysfunction, hepatic, or renal impairment. Serum bullous pemphigoid antibodies were absent. A clinical diagnosis of prurigo nodularis was made based on examination findings of excoriated nodules and the chronic, treatment-resistant nature of her itch. A punch biopsy of the left upper arm confirmed the diagnosis of PN (Fig 2).

Initial management included high-potency topical corticosteroids (mometasone and betamethasone dipropionate), oral antihistamines (cetirizine), and neuromodulators (gabapentin and duloxetine), none of which offered significant relief. She subsequently received a prednisone taper (50 mg daily for 4 days, then reduced by 10 mg every 4 days until discontinuation over 20 days) and a trial of aprepitant, all without improvement. A longer course of systemic corticosteroids was not prescribed given her lack of response and the associated risks given her history of diabetes. Traditional systemic immunosuppressants such as methotrexate and cyclosporine were deferred due to her active breast cancer treatment and concern for immunosuppression and drug interactions. Due to persistent symptoms, dupilumab (600 mg loading dose followed by 300 mg subcutaneously every 2 weeks) was initiated, given its potential efficacy and favorable safety profile. After 3 months without meaningful response, colchicine was added. However, the patient continued to experience disabling pruritus and associated decrement in her quality of life.

**Fig 3:**
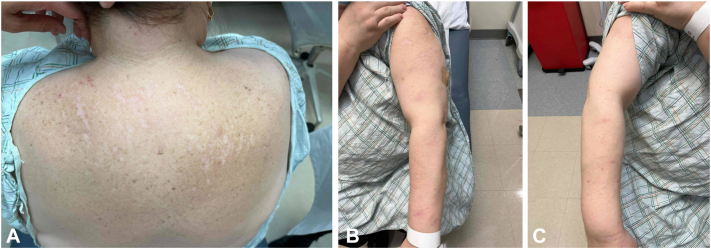
Patient achieved clearance after treatment with nemolizumab. There are **(A)(B)(C)** scattered linear scars with minimal erythema and postinflammatory pigmentation changes on the back and bilateral upper extremities along with self-reported decrease in pruritus symptoms and clearance of prior prurigo nodules.

Given the failure of multiple agents including IL4/IL13 blockade with dupilumab, nemolizumab was initiated. The patient received 3 monthly doses. Upon clinical follow-up, she reported 95% resolution of symptoms, with only residual postinflammatory changes on examination (Fig 3). She maintained sustained resolution of symptoms following these doses for an additional 4 months, and elected to restart monthly nemolizumab during subsequent flare of PN thereafter, again with good effect.

## Discussion

PN is a chronic, intensely pruritic dermatologic condition with a multifactorial pathogenesis that includes both immunologic and neurologic components. There is evidence recognizing it as a disorder of neuroimmune dysregulation, with IL-31 emerging as a key cytokine driving the associated pruritus, an association with increased dermal nerve fiber density, and upregulation of pruritogenic neuropeptides such as substance P and calcitonin gene-related peptide.^1^^,^^2^ Despite numerous available treatments, PN remains a therapeutically challenging disease, particularly in patients with underlying medical comorbidities or treatment-refractory symptoms.

This case represents a rare instance of trastuzumab-associated PN, with the patient's symptoms refractory to conventional antipruritic therapies and dupilumab.^5^ Temporally, her presentation was associated with the initiation of HER2-directed therapy; alternative etiologies such as anemia, hepatic or renal dysfunction, bullous pemphigoid, or drug hypersensitivity reactions were effectively excluded through laboratory and clinical assessment. Notably, her facial involvement, emergency department visit for uncontrolled symptoms, and lack of response to systemic neuropathic and anti-inflammatory agents underscore the burden of disease and added to her therapeutic complexity.

The mechanism of trastuzumab-associated pruritus remains poorly understood but may involve dysregulation of neuroimmune pathways, epidermal barrier function, photosensitivity, or immune-mediated cytokine release.^2^^,^^6^ IL-31 is a key pruritogenic cytokine secreted by activated T cells, and its receptor is abundantly expressed on cutaneous sensory neurons and keratinocytes, making it a logical target in cases of severe, unresponsive pruritus.^7^

Our patient failed a wide array of first- and second-line therapies, including topical corticosteroids, oral antihistamines, gabapentin, serotonin-norepinephrine reuptake inhibitors, systemic corticosteroids, and aprepitant. She was started on dupilumab, a biologic approved for PN in 2022, but experienced no clinical improvement over several months.^8^ Notably, the patient achieved substantial, sustained improvement only after initiating nemolizumab, a monoclonal antibody directed against the IL-31 receptor A, administered monthly.^3^ After approximately 3 doses, she reported near-complete resolution of her symptoms with >95% improvement in pruritus and skin lesions, sustained even after discontinuation of the drug.

This case supports growing evidence for the role of IL-31 blockade in PN, particularly in biologic-refractory or drug-induced disease. While nemolizumab received FDA approval for PN in 2024, literature describing its use in oncology-associated cutaneous toxicities remains limited.^3^ This case illustrates its potential benefit in trastuzumab-related pruritic dermatoses, expanding its application to iatrogenic PN in cancer patients. It also emphasizes the importance of integrating dermatologic expertise in oncologic care, particularly when cutaneous adverse effects interfere with quality of life or therapeutic adherence.

## Conflicts of interest

None disclosed.
